# How Do We Bridge the Gap Between Animal Models of Sepsis and Patients?

**DOI:** 10.34067/KID.0000000000000458

**Published:** 2024-05-30

**Authors:** Naoki Hayase, Kent Doi

**Affiliations:** Department of Emergency and Critical Care Medicine, The University of Tokyo, Tokyo, Japan

**Keywords:** acute kidney failure, AKI, acute renal failure

Sepsis accounts for more than 50% of hospital deaths in the United States, and the cost of sepsis treatment ranks at the top of the list.^1^ Unfortunately, we do not have definitive therapeutic strategies for sepsis beyond supportive care. Although there are numbers of preclinical studies that report positive results of promising drugs, most of the results obtained by animal sepsis models have failed to be translated to the clinical. For example, the effectiveness of anti–tumor necrosis factor-*α* therapy was proven in lipopolysaccharide-induced septic animals, whereas the drug against tumor necrosis factor-*α* failed to improve the survival of septic patients in the clinical trial.^2^ Why did that happen? Such translation failure could be mainly attributed to the disparity between animal sepsis models and patients.^3,4^

There are multiple possible reasons for this gap. The etiologies of human sepsis are more complex and multifactorial. In most septic animals, drug administration is often initiated before or a few hours after septic insults while septic patients are seldom treated in such early window.^3^ Among the reasons, it is notable that animal sepsis studies using healthy individuals cannot recapitulate human septic population. Reflecting the aging society, a growing number of patients in intensive care unit (ICU) present with one or more comorbidities. In addition, a recent epidemiological study showed that sepsis most frequently occurred among the patients with preexisting comorbid conditions such as CKD, liver disease, and diabetes mellitus with very high mortality in these patient population.^5^ It might be important to study sepsis in animal models complicated with preexisting chronic diseases. However, there have been few studies to address this reverse-modeling from bedside to bench because of its technical difficulty and labor-intensive process.

Two-stage mouse models of preexisting renal disease with subsequent cecum ligation and puncture (CLP)–induced sepsis have been developed.^6,7^ These CKD–sepsis models increased vascular permeability in the lung and blood proinflammatory cytokines and decreased bacterial clearance compared with the septic mice without CKD. Interestingly, soluble fms-like tyrosine kinase 1 (a soluble vascular endothelial growth factor receptor), which improved the survival of the CKD–sepsis mice, were ineffective in the septic mice without CKD. These findings suggested the septic animals with comorbidities have some distinct difference in underlying pathophysiological mechanisms of sepsis development from sepsis models in healthy animals.

In this issue of *Kidney360*, Floyd and colleagues reported a novel two-stage mouse model of sepsis complicated with preexisting acute kidney disease (AKD) or CKD to replicate the complexity and common clinical profile of septic patients.^8^ They induced experimental AKD and CKD using aristolochic acid (AA) and superimposed cecal slurry (CS) injection-induced sepsis on them. This study exceled at the following points; first, they developed stable AA-induced AKD and CKD with the small variation of GFR. Second, sepsis severity was successfully adjusted by titrating the dose of the CS to enable them to assess the longitudinal change of organ injury markers and immune response during the development of sepsis. CLP has been well recognized as a gold standard of sepsis animal model. However, CLP tends to cause high disease severity because it involves major surgery, prolonged anesthesia, and ischemic insult by ligation of cecum. On the other hand, CS implantation is the emerging strategy to induce polymicrobial sepsis. As they mentioned, it can generate dose-dependent sepsis severity and avoid unnecessary surgical and anesthetic insults.

It should be noted that AKD mice with subsequent sepsis had higher mortality and sepsis severity than CKD mice even though AKD mice received less cumulative amount of AA and higher GFR in this study. AKD–sepsis mice also showed distant organ injuries, decreased bacterial clearance, and acute elevation of proinflammatory and anti-inflammatory cytokines. The flow cytometry showed persistent significant neutropenia in those mice while less reduction of neutrophils was observed in CKD-sepsis mice. Currently, AKD and CKD are defined by different diagnosis criteria with different observation periods. AKD, including AKI, is defined by functional and/or structural abnormalities for ≤3 months. CKD is defined by markers of kidney damage or decreased GFR persisting for >3 months. Reportedly, the uremic state in advanced CKD and end-stage renal disease are also associated with impaired innate and adaptive immune system,^9^ which could contribute to the higher sepsis severity of septic mice with preexisting CKD than those without CKD. However, it is still obscure whether this immunological change caused poor prognosis of AKD-sepsis mice. It is also unclear why does the shorter period of renal dysfunction in AKD cause more severe organ dysfunctions and immunological disorders. In addition, the clinical relevance of AA could be limited because of the infrequency of AA nephropathy in human. Further research is warranted to elucidate underlying mechanism for higher mortality and severity of AKD–sepsis mice.

To increase clinical relevance of sepsis animal models, the following points should be considered. First, the difference in sex and age might affect the sepsis severity. Elderly patients are predominant in ICU patient population. Most of the animal sepsis studies involve only male patients, whereas female patients account for just less than 50% of septic patients.^10^ Second, it might be better to incorporate the treatments used in clinical ICU settings (*e.g*., antibiotics, source control, and sedation/analgesia). Third, their study results should be confirmed in the mouse models with sepsis superimposed on other chronic diseases such as diabetes and heart failure to increase generalizability. Finally, future multicenter preclinical studies could be warranted for overcoming limitations of a single-laboratory study.

In summary, previous failures of translation from the bench to the bedside in sepsis treatment underscores the unfilled gap between animal sepsis model and patients and the necessity to develop more clinically relevant sepsis models. Floyd and colleagues generated their novel mouse model of sepsis superimposed on preexisting kidney disease, suggesting that patients with AKD may be more vulnerable to subsequent sepsis than patients with CKD. This finding may indicate a novel mechanism of disease in sepsis, which can be a target for specific therapy against sepsis.
